# Enhancing generic capabilities and metacognitive awareness of first-year nursing students using active learning strategy

**DOI:** 10.1186/s12912-021-00601-7

**Published:** 2021-05-22

**Authors:** Carmen Wing Han Chan, Fiona Wing Ki Tang, Ka Ming Chow, Cho Lee Wong

**Affiliations:** The Nethersole School of Nursing, Faculty of Medicine, The Chinese University of Hong Kong, Shatin, NT Hong Kong

**Keywords:** Active learning, Generic capabilities, Metacognitive awareness, Nursing students

## Abstract

**Background:**

Developing students’ generic capabilities is a major goal of university education as it can help to equip students with life-long learning skills and promote holistic personal development. However, traditional didactic teaching has not been very successful in achieving this aim. Kember and Leung’s Teaching and Learning Model suggests an interactive learning environment has a strong impact on developing students’ generic capabilities. Metacognitive awareness is also known to be related to generic capability development. This study aimed to assess changes on the development of generic capabilities and metacognitive awareness after the introduction of active learning strategy among nursing students.

**Methods:**

This study adopted a quasi-experimental single group, matched pre- and posttest design. It was conducted in a school of nursing at a university in Hong Kong. Active learning approaches included the flipped classroom (an emphasis on pre-reading) and enhanced lectures (the breaking down of a long lecture into several mini-lectures and supplemented by interactive learning activities) were introduced in a foundational nursing course. The Capabilities Subscale of the Student Engagement Questionnaire and the Metacognitive Awareness Inventory were administered to two hundred students at the start (T0) and at the end of the course (T1). A paired t-test was performed to examine the changes in general capabilities and metacognitive awareness between T0 and T1.

**Results:**

A total of 139 paired pre- and post-study responses (69.5 %) were received. Significant improvements were observed in the critical thinking (*p* < 0.001), creative thinking (*p* = 0.03), problem-solving (*p* < 0.001) and communication skills (*p* = 0.04) with the implementation of active learning. Significant changes were also observed in knowledge of cognition (*p* < 0.001) and regulation of cognition (p < 0.001) in the metacognitive awareness scales.

**Conclusions:**

Active learning is a novel and effective teaching approach that can be applied in the nursing education field. It has great potential to enhance students’ development of generic capabilities and metacognitive awareness.

## Background

A university education should go beyond the delivery of knowledge in a particular subject to equip students of all disciplines and professions with the necessary skills for societal and professional sustainability [1, 2]. According to Kember [1], these necessary skills or attributes are generally referred to as generic capabilities and include creative thinking, self-managed learning, problem solving, adaptability, communication skills, interpersonal skills and group work.

The undergraduate nursing curriculum in many places such as Hong Kong is discipline-driven: strong theoretical knowledge, decision-making and clinical reasoning are vital attributes for nursing practice. Teachers are very used to applying the long-standing tradition of highly didactic teaching. Although this method can disseminate a large amount of information, it offers few opportunities for feedback, student engagement, peer interaction, or the application of knowledge [3]. Very often, the students are moulded into passive learners by didactic lectures [4]. Another characteristic of didactic lecturing is the simple faith that students can learn and understand what they are told. However, educational experts have provided new insights that suggest successful learning is a much more complex process than just listening [4].

Research evidence suggests that students adopt various learning strategies such as metacognition, time management and effort regulation to improve their academic outcomes [5]. Metacognition is defined as the knowledge and control of learning strategies by perceiving to what degree individuals are aware of these learning strategies, how they understand them to work, and how they know when to use them [6, 7]. Metacognition has been found to facilitate students’ learning performance and the development of their generic capabilities [8–11]. Various studies have demonstrated that university students with a higher level of metacognitive awareness have better decision-making skills [6] and achieve better academic performance [12, 13]. Students with poor metacognitive awareness usually employ ineffective learning strategies, and eventually fail to make use of their thinking processes or to develop practical skills to overcome learning challenges [9, 14].

The nature of the educational process and its environment have a strong impact on the development of generic capabilities [1]. An effective teaching and learning environment influence the development of seven generic capabilities: critical thinking, creative thinking, self-managed learning, adaptability, problem-solving, communication skills, and interpersonal skills and group work [1, 15]. The principal mechanism of capability development is largely based on a teaching and learning environment characterised by the active participation of students in learning activities, and a high degree of teacher–student and student–student interaction [15].

Incorporating an active learning approach in teaching is indicated to engage students and increase their active participation in learning [3]. Petress [16] defined active learning as “*a process where the learner takes a dynamic and energetic role in one’s own education*”. The use of active learning has also been reported to improve teacher–student interaction and the attitudes of students towards learning [17], as well as students’ interest in learning and self-learning abilities [18]. The flipped classroom and enhanced lectures [4, 19] are two examples of active learning approaches. The flipped classroom implies inverting the expectations of a traditional one-way lecture. It affirms the importance of lectures and assignments but the position and sequence are flipped [19–21]. This affirmation is important, so that adopting such a change is less challenging to nursing teachers’ traditional values when giving lectures. The underlying imperative is that students gather most of the information before class by reading, researching information, or watching recorded lectures [20]. The purpose of the flipped classroom is to provide students with an opportunity to view course content in their own time before the lecture, which results in a more efficient use of students’ time during the class itself [19–21]. Pre-class learning materials (such as video-taped lecture, role-play videos, reading, exercises, or quizzes) work best when teachers tailor-make them for each class. However, they are not a replacement for lectures, but they do allow students to prepare for the class and open up the time in the classroom for engagement in problem-solving and interactive activities [19, 20].

Previous studies have shown that the use of flipped classroom in various disciplines in universities may be effective in promoting students’ learning outcomes such as engagement, metacognition, attitude, motivation, and performance [22, 23]. In the past decade, there has been growing interest for nursing educators to adopt the flipped learning approach in response to the increasing complexity of nursing care. In a systematic review of five studies that investigated the use of flipped classroom in higher education nursing programmes, the authors conclude that this style of teaching yield neutral or positive academic outcomes and mixed findings for student satisfaction [24]. More recently, a meta-analysis of 32 randomised control trials conducted in China indicated that the flipped classroom approach, compared to the traditional lecture based approach, produced significantly higher theoretical scores and skills scores in Chinese nursing students [25]. In view of these encouraging results, further studies are warranted to confirm the benefits of adopting the flipping learning approach in nursing education.

An enhanced lecture is a series of mini-lectures supplemented by active learning activities [4]. The purpose is to maximise learning and allow students to become active learners to take charge of their own learning [21]. Discussions, short writings, lecture summaries, or quizzes can provide timely feedback about the extent of student learning. Activities also maintain students’ attention [4]. A higher level of engagement in effective learning strategies, such as collaborative or self-directed learning, has also been observed among students who have become active learners [4, 26]. Moreover, research has shown that an active learning approach and environment can help to encourage the development of metacognitive awareness in undergraduate nursing students [27, 28].

Nevertheless, studies that evaluated the effects of active learning on the development of generic capabilities and metacognitive awareness in nursing students are seemingly lacking. Therefore, this study aimed to evaluate the effects of active learning on the development of generic capabilities and metacognitive awareness in nursing students. The objectives were: (1) To develop and produce teaching materials for active learning; (2) To implement active learning approaches in classroom teaching during a first-year nursing course; and (3) To assess changes in student outcomes in metacognitive awareness and generic capabilities after implementing active learning approaches. We hypothesized that nursing students’ metacognitive awareness and generic capabilities would be enhanced after implementing active learning approaches.

## Methods

### Design

A quasi-experimental single group, matched pre- and post-test design was adopted.

### Subjects and setting

A convenience sample approach was adopted to recruit first-year Bachelor of Nursing students of the study institution. Since the active learning approaches were incorporated in the Fundamentals of Nursing I course, the two hundred Year 1 students who enrolled this course were eligible to participate in the study.

### Procedures

#### Phase 1: planning

The planning phase aimed at developing an integrated approach to embed active learning in the teaching and learning environment. The use of active learning approaches in classroom teaching requires a flexible environment, a shift in learning culture, well-selected teaching materials, and skilled and well-trained educators [21]. Teachers need access to research evidence as well as teaching and learning resources to implement active learning. The teacher’s role in a classroom shifts from instruction to observation, feedback, guidance, and assessment. For this reason, a workshop was held for all teachers in the nursing school to promote active learning. The contents of the workshop included the major characteristics of the approach, the strengths and weaknesses of didactic lectures versus active learning, obstacles or barriers which prevent teachers and students from using active learning strategies, and solutions to overcome these barriers.

In addition to this, several teaching team meetings were held before the course commenced to determine how active learning could be implemented. Another purpose of these meetings was to discuss the production of new teaching materials, such as videos, scenarios, recorded lectures, e-learning modules, discussion questions, quizzes and other in-class activities that might enhance active learning. Hands-on experience with technology, for example handheld devices such as crickets, was also discussed during the meetings, as the use of technological inventions could help to promote on-site interaction between teacher and students as well as between students themselves [29]. Informal feedbacks from the teachers indicated that the workshop was useful. Their concerns related to the implementation of flipped classroom, such as ways to motivate students to read the pre-class materials or engage in class discussion, were discussed during the workshop, which provide them with more confidence in adopting this innovative teaching approach. On the other hand, the teaching team meetings also provided them platform to share ideas on how to implement active learning effectively.

During the course introduction, students were told that they would be attending innovative and interactive lectures instead of the traditional didactic type. The expected learning activities were also outlined, and students were reminded to study the pre-class learning materials before attending classes.

#### Phase 2: implementation

The implementation of active learning was begun in a first-year nursing course as it was thought easier to build up a culture of active learning when students were relatively new to the programme. The course, Fundamentals of Nursing I, a three-unit credit course taught in year 1, was chosen for this study. This term 2 course usually commences at January and end at May each year. The assessment was based on a two-hour written examination (50 %), a skill competency examination on vital signs (30 %), and three web-based quizzes (20 %). The course was taught by five faculty members. The course adopted a lecture and e-learning format, consisting of two one-hour lectures, two one-hour tutorial/laboratory sessions and one to two hours of e-learning each week over 13 weeks. In previous years, lectures had tended to be primarily the one-way transfer of information in a traditional format, and were all conducted in a lecture theatre with fixed, non-movable rows of seating, and one large video screen at the front of the room.

For this study, the course was redesigned to incorporate active learning approaches, including flipped classroom and enhanced lecturing in selected lectures. Five out of 13 lectures were selected because a previous study had found that students did not like all their classes to be flipped [30]. The topic of the five lectures were “vital signs”, “meeting safety needs I”, “meeting safety needs II”, “infection control related to nursing” and “process of wound healing”, selected because they contained a mixture of nursing knowledge, theories, and skills and were more appropriate for making videos and scenarios than other lectures in this course. Pre-class learning materials (such as PowerPoint slides, videos, and journal excerpts) were tailor-made for each class. With these five selected topics, approximately 30 % of the class time was spent on mini-lectures, and the remaining 70 % on guided discussion.

Students were required to read or view pre-class learning materials before attending classes to obtain a brief overview of the fundamental concepts that would be covered in the lecture. During the first 15 min, the teacher would review all the important points presented in the pre-class materials. Students were then divided into pre-assigned smaller groups (with 15–18 people in each) and worked collaboratively to discuss case scenarios or critique videos. This session usually lasted between 30 and 45 min to allow the students to share ideas and learn from their peers. Afterwards, one to two representatives from each group would be invited to present the group’s findings to the class. Following the presentation, the teachers provided feedback to the students. A short quiz would sometimes be set to help consolidate students’ knowledge. Finally, the teachers presented a short (but in-depth) summary of the topic. Table 1 summarises the course format changes.

**Table 1:** The changes of course format

Topic	Content	Learning materials prepared or developed
Vital signs	Factors affecting vital signs and accurate measurement of them.	-A tailor-made video demonstrating skill in vital signs taking. -A tailor-made video for students to critique good or poor practice in vital signs taking. -In-class quizzes
Meeting safety needs I	Care of clients requiring special protection and care.	-Case scenarios were developed for students to discuss factors contributing to falls in the elderly.
Meeting safety needs II	Care of the very ill, the delirious/ restless client, and use of safety devices.	-Case scenarios were developed for students to discuss nursing interventions that prevent falls in hospital and home setting. -10-item online quizzes.
Process of wound healing	Wound healing process and basic wound care.	-Case scenarios were developed for students to discuss factors that promote or delay wound healing. -Discussion of nursing interventions that promote wound healing. -In-class quizzes
Infection control in relation to nursing	Principles of aseptic techniques and nursing management for clients with acute and chronic wounds.	-Different wound pictures were prepared for students to discuss types of wounds and the use of different dressing materials. -A tailor-made video demonstrating skill in wound dressing. -A tailor-made video for students to critique good or poor practice in wound dressing.

#### Phase 3: monitoring

This phase involved monitoring the results and gathering feedback from participants. Three questionnaires, the Capabilities Subscale of Student Engagement Questionnaire (SEQ), Metacognitive Awareness Inventory (MAI) and a brief demographic sheet (age, gender and residence), were employed.

The capability subscale of the SEQ developed by Kember & Leung [15] was used to collect data about students’ reflections on the development of their generic capabilities. It consists of 16 items measuring eight aspects of capability: critical thinking (2 items), creative thinking (2 items), self-managed learning (2 items), adaptability (2 items), problem-solving (2 items), communication skills (2 items), interpersonal skills and group-work (2 items), and computer literacy (2 items). The responses were recorded on five-point Likert scale ranging from 5 (‘strongly agree’) to 1 (‘strongly disagree’). The SEQ has demonstrated good psychometric properties in Hong Kong undergraduate samples [15]. The questionnaire also contains two open-ended questions to gather feedback on the best aspect and that most in need of improvement. In this study, the reliability of the capability subscale was high, with Cronbach’s alphas of 0.86 and 0.92.

The MAI developed by Schraw & Dennison [14] is a 52-item, self-reported questionnaire designed to investigate adults’ metacognitive awareness. It consists of two subscales: knowledge of cognition (17 items) and regulation of cognition (35 items). Knowledge of cognition involves awareness of one’s personal strengths and weaknesses in learning, knowledge about learning strategies, and why and when those strategies should be used. The regulation of cognition subscale measures knowledge about planning, implementing, monitoring and evaluating the learning strategies that are being used. The items are rated on a five-point Likert-type scale, from 1 (‘always false’) to 5 (‘always true’). In this study, the reliability of the two subscales was high, with Cronbach’s alphas of 0.80 and 0.96.

### Data collection and management

During the first lecture all students were provided with study consent forms and questionnaires and invited to complete at the start (T0) an evaluation of the capability subscale of SEQ and MAI, and again at the end (T1) of the course when active learning sessions were completed.

SPSS version 24.0 (SPSS, Chicago IL) was used for data analysis, and descriptive statistics to present participants’ characteristics and capability and MAI scores at T0 and T1. Shapiro-Wilks test was used to test for normality of continuous variables. A paired t-test was performed to compare the differences in the change of the outcome variables (T0–T1). The level of statistical significance for all analyses was set at 5 %.

## Results

### Participant characteristics

A total of 139 paired pre- and post-study responses (69.5 %) were received. The mean age of the participants was 18.74 (SD = 0.97), the majority (78.4 %) were female and nearly half lived in a campus hall of residence (48.9 %).

### Generic capabilities

Table 2 shows the mean and standard deviations of the capability scores as rated by the participants. Among the eight generic capabilities, the participants reported significant improvements in their critical thinking (*p* < 0.001), creative thinking (*p* = 0.03), problem-solving (*p* < 0.001), and communication skills (*p* = 0.04) by the end of the course.

**Table 2:** Descriptive and comparison summary of the capability scores

	Pre-test Mean (SD)	Post-test Mean (SD)	*p*-value
**Capabilities**			
Critical thinking	3.82 (0.39)	3.96 (0.47)	< 0.001
Creative thinking	3.74 (0.53)	3.86 (0.56)	0.03
Self-managed learning	3.86 (0.52)	3.86 (0.60)	0.95
Adaptability	3.92 (0.44)	4.00 (0.55)	0.11
Problem solving	3.76 (0.47)	3.96 (0.54)	< 0.001
Communication skills	3.77 (0.47)	3.90 (0.62)	0.04
Interpersonal skills & group work	3.78 (0.50)	3.82 (0.60)	0.50
Computer literacy	3.71 (0.56)	3.73 (0.75)	0.84

Note: A paired t-test was performed, with p < 0.05 considered statistically significant

### Metacognitive awareness

Table 3 shows the mean and standard deviations of the MAI scores as rated by the participants. The overall scores in the MAI scales (*p* < 0.001), together with the knowledge of cognition subscales (*p* < 0.001) and regulation of cognition subscales (*p* < 0.001), were significantly higher at the end of the course, which indicated significant improvements in the participants’ metacognitive awareness.

**Table 3:** Descriptive and comparison summary of the metacognitive awareness scores

	Pre-test Mean (SD)	Post-test Mean (SD)	*p*-value
**Knowledge of Cognition subscale**			
Declarative knowledge	28.87 (3.26)	30.35 (3.42)	< 0.001
Procedural knowledge	14.68 (1.60)	15.42 (2.84)	< 0.001
Conditional knowledge	18.16 (2.21)	19.07 (2.01)	< 0.001
Total	61.71 (6.60)	64.84 (6.95)	< 0.001
**Regulation of Cognition subscale**			
Planning	25.50 (2.96)	26.62 (4.58)	< 0.001
Information management	36.74 (4.08)	38.58 (5.11)	< 0.001
Monitoring	25.53 (2.90)	26.31 (2.83)	< 0.001
Debugging	18.61 (2.40)	19.36 (2.05)	< 0.001
Evaluation	21.68 (2.82)	22.63 (2.48)	< 0.001
Total	128.05 (13.76)	133.49 (13.70)	< 0.001
**MAI total**	189.76 (20.04)	198.33 (19.67)	< 0.001

*MAI* Metacognitive Awareness Inventory; A paired t-test was performed with *p* < 0.05 considered statistically significant

### Responses to two open-ended questions

A total of 86 students provided their responses to the two open-ended questions. A majority of the students commented that the teaching materials were good/ great/ excellent (*n* = 67), quite sufficient and rewarding (*n* = 48). They also found that the lectures were well-planned (*n* = 35), practical (*n* = 32) and flexible (*n* = 12). However, some students (*n* = 26) thought that the lecturers should manage the time better. Additionally, a few students (n = 9) reported having difficulty in concentrating in class, and the need for further clarification of certain concepts.

## Discussion

As the undergraduate nursing curriculum is discipline-driven, the application of an active learning approach may not necessarily replace all traditional lectures. Findings of the present study support the hypothesis and the proposition that supplementing didactic lectures with active learning could enhance the generic capabilities and metacognitive awareness of students.

Students’ self-perceived critical thinking, creative thinking, problem-solving and communication skills all significantly improved after the implementation of the active learning approach, indicating that active learning could significantly enhance the development of students’ generic capabilities. These findings echoed a previous study that showed these capabilities could be enhanced by immersion in a stimulating and active environment that required students to practise their capabilities [15]. Our finding of improved critical thinking skills is in agreement with the finding by Dehghanzadeh & Jafaraghaee [31], who found a positive effect of flipped classroom on Iranian nursing students’ critical thinking disposition.

The implementation of the flipped classroom increased the responsibility of students for their own learning and gave them additional flexibility in the process [32]. Reading pre-class learning materials helped the students understand basic nursing concepts at their own pace and improved mastery of the course content, which could not possibly have been achieved in a traditional lecture setting [33]. In this study, teachers were able to maximise the class time by two means: (i) engaging students in different interactive and collaborative learning activities, such as case discussions, presentations, video critiques and in-class quizzes; and (ii) providing them with timely and constructive feedback in a face-to-face setting. Misconceptions could be corrected and enquiries pointed in the right directions. The flipped classroom helped students apply what they had learnt at a new level of understanding and engage them in higher-order thinking, such as analysis, synthesis, application and evaluation [20, 21]. In this way, it helped to develop students’ critical thinking and problem-solving skills [20]. In the same way, discussing case scenarios in class not only helped promote interaction among the students, but also improved their communication skills, stimulated peer-to-peer learning and, therefore, promoted creative thinking [34]. In short, the systematic incorporation of brief activities in class countered the many limitations of didactic lectures where students are not actively engaged in processing information or developing an understanding of the required information [35, 36].

Although it is not an objective of this study to evaluate academic performance, the course results also reflected positive changes when compared with previous cohorts where the traditional didactic methods had been used. Specifically, the assessment performance of this cohort was slightly better than the previous cohort (unpublished data). This finding is consistent with the findings of similar studies in China [25].

In this study, student perception of self-managed learning, adaptability, computer literacy, interpersonal skills and group work showed slight improvement but were not significant, which might be attributed to the fact that the selected course focused more on hard and fast nursing knowledge, skill and rationality. In addition, developing these capabilities would probably take a longer period of immersion in a university learning environment rather than just a 13-week foundation course.

The open-ended comments of the participants indicated that the course was well-planned and flexible, and also reflected their appreciation of the teaching and learning environment. In fact, a higher rating on satisfaction was obtained on the course and teaching evaluation in this cohort when compared with the previous cohort (unpublished data). However, a few students commented that they could not concentrate in class and that they needed further clarification of key concepts. This was understandable – the students had no prior knowledge of nursing, and the teacher therefore needed to be more aware of their individual differences in intellect, personality or lifespan role development. This further supported the emphasis on timely feedback and active student engagement [1], so that those students in need could be identified.

The positive result in metacognitive awareness is in line with previous studies showing that better awareness of the learning strategy is associated with better generic capabilities resulting from an active and conducive learning environment [6, 27]. The interactive learning activities provided in the course produced a learning environment that enabled students to plan, organise, implement and evaluate their own learning strategies. This learning process included the crucial elements of cognition regulation [14]. The activities also required the students to search for information and explore alternatives to improve their nursing practice. They were thus guided to think inductively, which was crucial to enhancing their metacognition [37, 38]. Our newly designed course provided positive indication that active learning could help to develop the metacognitive awareness of first-year nursing students.

## Limitations

This study has several limitations. First, no control or comparison groups were used, and so, any causal relationship between active learning and the outcomes could not be tested. Future study may adopt a historically controlled design, in which the outcome measure of Year 1 students (without flipped learning as control) will be compared with that same cohort of students who will be provided with flipped learning (intervention) when they move to Year 2. The advantage of this study design is fairness and minimal risk of contamination. Second, the self-reported questionnaires had inherent limitations, such as subjectivity, although such instruments are commonly used for assessing outcomes in educational research. Third, the cross-sectional design might not capture longer-term changes in the generic capabilities and metacognitive awareness of the students. Future studies might consider adopting a longitudinal design and objective outcome measures, such as academic results and clinical decision-making. Finally, the response rate of this study was only 69.5 %, which could be attributed to the reluctance of some students to answer all the items in the questionnaire. Moreover, given that the questionnaires were administrated after the lecture, some people might have been unwilling to stay behind and spare the time to complete them. An online version of the questionnaire might be used in future to provide students with an additional option and to encourage them to complete the questionnaire [39].

## Conclusions

The active learning approach is a contemporary, innovative and effective way to enhance the learning process and to engage students in higher levels of learning. This is the first study to implement active learning in a foundation nursing course and to monitor its effectiveness in revealing the development of generic capabilities and metacognitive awareness among Hong Kong nursing students. Results showed that active learning could be applied in nursing studies to enhance students’ development of these areas. Significant improvements were observed in the critical and creative thinking, problem-solving and communication skills of the students after the implementation of active learning approaches, and significant changes were also observed in their metacognitive awareness. The findings of the current study could help motivate teachers to adopt the active learning approaches in other nursing courses. Further studies employing a longitudinal design and objective outcome measures are warranted to confirm the benefits of adopting active learning approaches in nursing education.

## Data Availability

The datasets used and/or analysed during the current study available from the corresponding author on reasonable request.

## Abbreviations

MAI: Metacognitive Awareness Inventory
SEQ: Student Engagement Questionnaire
